# Serum brain-derived neurotrophic factor (BDNF) and self-paced time-trial performance in older untrained men

**DOI:** 10.1371/journal.pone.0285628

**Published:** 2023-07-03

**Authors:** Eevon Chia, Frank E. Marino

**Affiliations:** School of Allied Health, Exercise & Sports Science and Research Group for Human Adaptation, Exercise & Health, Charles Sturt University, Bathurst, NSW, Australia; University of Sao Paulo: Universidade de Sao Paulo, BRAZIL

## Abstract

**Purpose:**

This study examined the effect of 12 weeks of concurrent aerobic and resistance training on brain derived neurotrophic factor (BDNF) levels, neuromuscular performance and cerebral oxygenation on self-paced cycling exercise in previously untrained older men.

**Methods:**

Eight untrained healthy males aged 53–64 years performed a familiarisation and a pre-training self-paced cycling time trial before 12 weeks of exercise training which combined aerobic and resistance exercise. The self-paced cycling time trial comprised a 30 s maximal effort sprint for every 4.5 min of lower intensity pace for a total of 25 min. Upon completion of 12 weeks of training, a comparison of the pre-training trial analysed for serum BDNF, neuromuscular performance, and cerebral oxygenation was undertaken.

**Results:**

Serum BDNF decreased significantly from 10.02 ± 4.63 to 6.96 ± 3.56 ng/ml after 12 weeks of training. There was also attenuated physiological strain for a comparable self-paced cycling performance. Despite positive physiological responses during the time trial pacing strategy was not altered compared with pre training.

**Conclusion:**

BDNF decreases following 12 weeks of concurrent training and might reflect neuroplasticity for this type of training stimulus. Exercise training in previously sedentary older men can result in a multitude of physical benefits, which may also confer a neuroprotective effect. However, specific training is required to improve pacing strategies in previously untrained older males.

**Clinical trial registration:**

Australian New Zealand Clinical Trials Registry number ACTRN12622001477718.

## Introduction

Aging is characterised physiologically by declines in, but not exclusively, the cardiovascular [1] and musculoskeletal systems [2]. These age-related physiological changes can also be associated with disease, disability, and ultimately death. However, the benefits of physical activity in attenuating the negative impact of aging are well-documented [3, 4]. Beginning in the third decade of life, sarcopenia can escalate in late middle-age which may be accompanied by a loss of function and increased disability, especially with decreased physical activity [2]. The decline in cardiovascular capacity and sarcopenia can account for some of the decrease in physical performance, but older athletes have exhibited declines in strength with maintenance of muscle cross-sectional area [5]. Thus, other factors such as declines in central motor drive, reduced peripheral neuromuscular performance [6], and/or altered muscle morphology [7] may also contribute to exercise performance decrements.

An additional component to consider is the apparent cognitive decline that occurs with aging, particularly as we approach the sixth decade of life [8]. There are many aspects of exercise which require cognitive capabilities especially with respect to decision making and tactical responses [9]. Evidence now indicates that endurance pacing is a cognitive capability [10] and that decisions about how to apportion energy during performance is critical. This is a key consideration since exercise at a self-chosen pace in some part relies on the cognitive decision- making processes as shown in school children [11] and athletes [12]. Presumably this would also be the case with older adults, but empirical evidence in this regard is lacking. However, in our previous companion study we reported that following 12 weeks of mixed training (aerobic and resistance), older men were able to modulate their pacing strategy in self-regulated cycling in the heat (32°C, 68% relative humidity (%*Rh*)) [13]. In contrast, the present observational study reports on the effect of similar training on time trial performance in moderate ambient conditions (21.8°C, 51% *Rh*). Although there is now evidence indicating that endurance pacing is affected by impaired cognitive abilities [14], to our knowledge, this cognitive based capability has yet to be studied in either trained or untrained older adults. If endurance pacing is thought to be reliant to some degree on cognitive capabilities, it follows that central changes which affect neuronal maintenance and growth would be an integral part of this process. This is an important consideration since older adults also compete in organised competitions or engage in regular recreational physical activity which is encouraged to maintain a healthy lifestyle.

Neurotrophins are a family of proteins that exert actions on the cells of the nervous system by way of maintenance and plasticity [15]. A particular neurotrophin molecule, brain derived neurotrophic factor (BDNF), is known to modulate synaptic plasticity [16] and plays a role in exercise, whereby, increases in metabolic rate are interfaced with changes in cognition. Evidence strongly suggests that regular exercise increases BDNF levels and can, therefore, maintain brain function and promote brain plasticity [3, 17]. Although it has not been specifically shown that BDNF alters performance of physical tasks where decision making is required, such as appropriate pacing, there is evidence to suggest that BDNF is influential in decision making more generally [18] and that acute and habitual aerobic exercise increases the level of peripheral BDNF, but little evidence exists that BDNF increases with resistance training [19]. To our knowledge there is no evidence that concurrent aerobic and resistance exercise alters BDNF levels, but presumably we would expect increases with this type of training. As such, the expectation would also be that concurrent aerobic and resistance training would improve pacing in self-regulated exercise given the potential cognitive component.

Near-infrared spectroscopy (NIRS) is a non-invasive technique used to evaluate changes in tissue oxygenation and monitoring cerebral oxygenation and haemodynamics during functional brain activation. Changes in oxyhaemoglobin concentration [O_2_Hb] measured by NIRS is an index of cerebral blood flow and reflects cortical activation during motor tasks [20]. In the muscle, NIRS oxygenation can be used to detect blood flow and provide an indication of O_2_ saturation [21] and deoxygenation reflects the balance between O_2_ delivery and utilisation [22]. NIRS also allows continuous measurement of [O_2_Hb] and deoxygenated haemoglobin [HHb] during task performance [23] and has been previously employed to assess prefrontal neuronal recruitment associated with exercise and pacing [24]. In addition to potential changes in cerebral oxygenation, it is also well documented that chronic exercise training will lead to improved neuromuscular performance as evidenced by increases in neural drive [25].

Therefore, the purpose of this study was to examine the effect of concurrent aerobic and resistance training on BDNF levels and whether this change would be accompanied by enhanced pacing strategies in previously untrained older men. A secondary aim was to examine whether changes in cerebral oxygenation and neuromuscular performance would be enhanced by chronic training in older males. We hypothesised that chronic training would increase circulating BDNF values and modulate cerebral oxygenation and accordingly there would be improved pacing during a self-paced time trial performance.

## Methods

### Study registration

**Fig 1:**
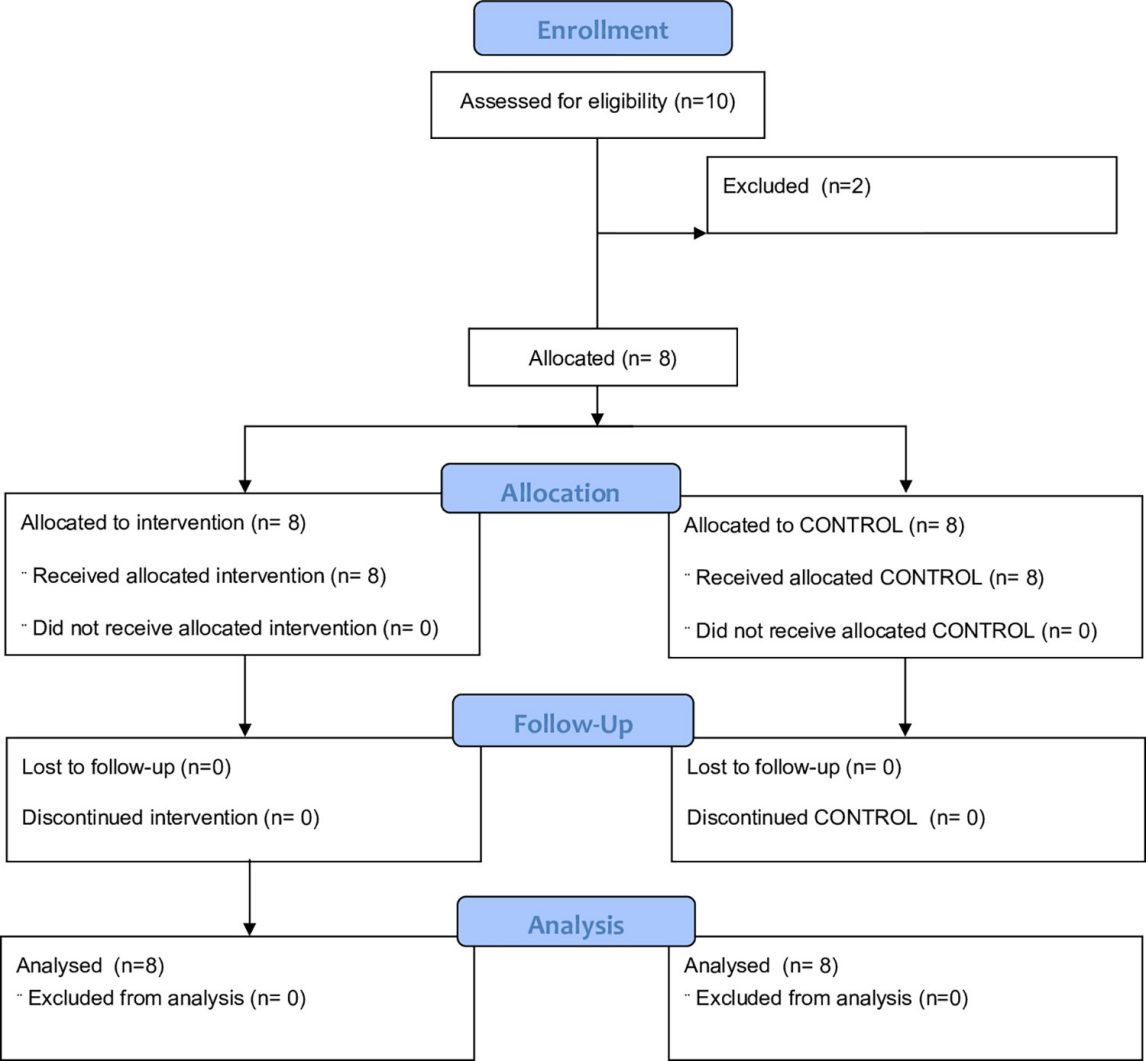
CONSORT flow diagram showing the enrolment, allocation and follow-up for the participants.

The study was retrospectively registered with the Australian New Zealand Clinical Trials Registry (ANZCTR; Registration number ACTRN12622001477718). The trial was retrospectively registered as researchers did not consider that predicated outcomes would have clinical value. Rather, the study was originally intended to refine measures of exercise performance with responses for BDNF only as a secondary outcome. The authors confirm that no further trials which include the measurement of BDNF have been conducted and no further trials relating to this intervention will be conducted. The CONSORT flow diagram relating the recruitment, allocation and follow-up is provided as Fig 1.

### Participants

Eight sedentary males aged (mean ± SD) 58.6 ± 4.2 years who had not participated in any regular exercise for more than 6 months were recruited from the local community between the months of July and January. They were all healthy non-smokers and were free from any known pathology or injury. The participants were classified as untrained according to the performance levels described by De Pauw et al. [26] which range in classification from 1–5 (untrained, recreationally trained, trained, well-trained, and professional). The study was approved by the Human Research Ethics Committee (HREC) of Charles Sturt University. After a thorough verbal and written explanation of the study, all participants were provided an opportunity to ask any questions, after which they signed a written letter of informed consent that was pre-approved by the HREC. There were no adverse effects observed or reported during the course of the experiment and there were no participant drop-outs.

### Study design

**Fig 2:**
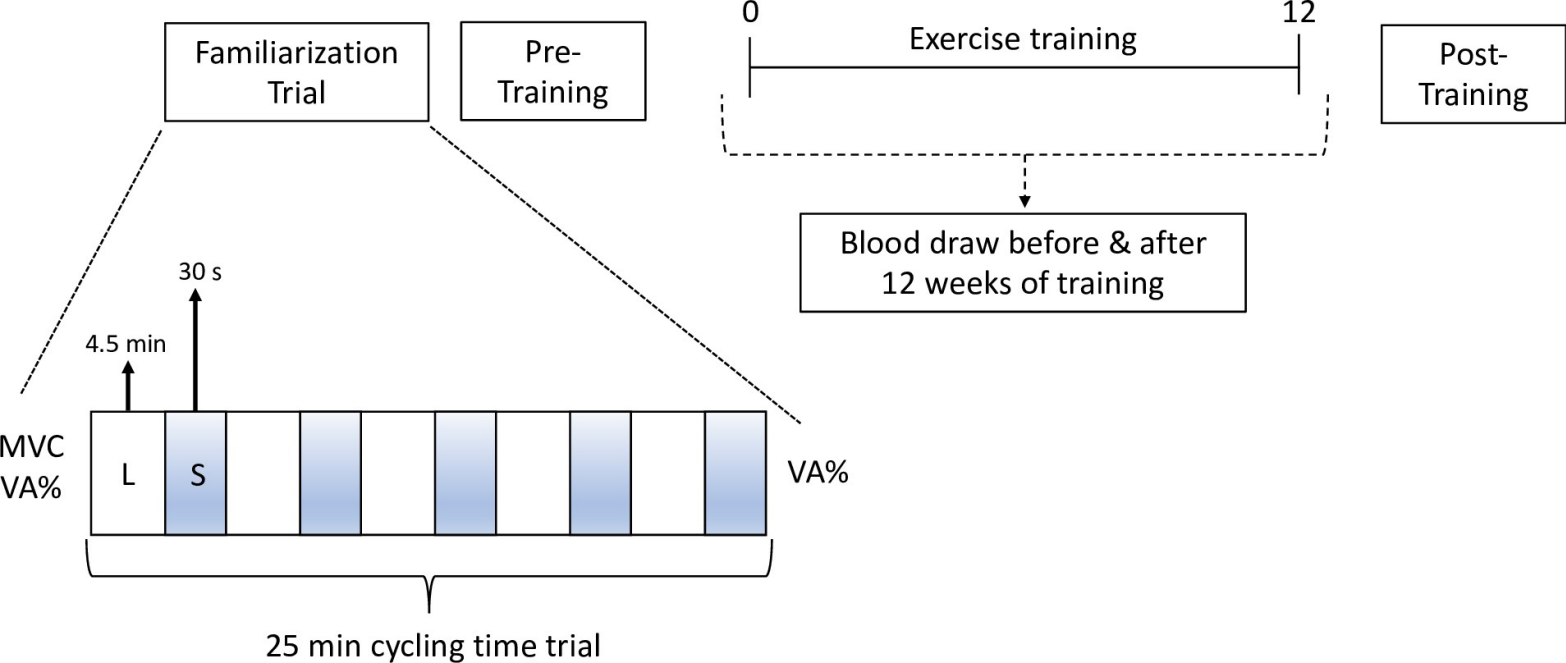
Schematic of study design. MVC = maximum voluntary contraction; VA% = level of voluntary activation; clear box labelled L represents 4.5 min of cycling at lower intensity; shaded grey box labelled S represents 30 s of maximal sprint effort.

The study design is shown in Fig 2. Participants attended two laboratory trials before commencing exercise training. The experiment was a randomised cross-over design where participants were randomly assigned by the chief investigator to the initial trial and then counterbalanced to minimise ordering effects. The protocol has been previously described in detail in a companion paper [13]. On the first occasion (familiarisation trial), each participant was familiarised with the cycling time trial which comprised a 30 s maximal effort sprint for every 4.5 min of lower intensity pace for a total of 25 min. Although the participants self-selected the gears and cadence throughout the entire 25 min, the time trial required them to cover as much distance as possible and be fully expended at the end of 25 min, i.e., the pace selected during the 4.5 min should have been one that was maximally sustainable for the duration keeping in mind the 30 s of maximal effort immediately following. Participants remained seated throughout the protocol. After 7–10 d, the participant returned to repeat the protocol. The level of physical activity was kept constant through a 1-week activity diary logged at baseline and used to remind participants before subsequent trials.

All trials were conducted on a cycle ergometer (Velotron Pro, RacerMate Inc., Seattle, WA) which was calibrated with the Accuwatt™ test (Velotron Coaching Software, Version 1.6.458, RacerMate Inc., Seattle, WA) before each trial. The saddle and handlebar positions were adjusted to fit each participant and the settings were recorded for subsequent trials. All trials were performed at the same time of the day in a climate controlled chamber with a mean dry bulb temperature and relative humidity of 21.8°C and 51%, respectively (Questemp° 15 Area Heat Stress Monitor, Quest Technologies, Oconomowoc, WI). Before each trial, each participant was asked to keep their eating habits consistent, aided by a food diary before the pre-training trial. Following the pre-training trial, the participants completed 12 weeks of exercise training (described subsequently). Upon completion of the last training session, participants returned to the lab to perform the cycling protocol. The mean delay between the last training session and performing the cycling protocol was 6 days.

### Height and body composition

During the familiarisation session, height was recorded in cm (S+M Height Measure, Aaxis Pacific, NSW, Australia), followed by supine whole-body dual-energy X-ray absorptiometry (DXA) scan for analysis of whole body and regional body composition (Norland XR-800, CooperSurgical Inc., CT). Scanning resolution was set at 6.5 × 13.0 mm and scanning speed was set at 130 mm/s (Illuminatus DXA™ User Interface Software Version 4.2.0). The whole body was analysed for lean & fat mass in kg and percentage and the right thigh was analysed for lean mass to calculate muscle quality using Eq 1:

### Brain-derived neurotrophic factor (BDNF)

A 4-ml blood sample (VACUETTE® Z Serum Clot Activator) was collected via aseptic venipuncture at the same time of the day for each participant from the antecubital vein while resting in a seated position for the determination of resting serum BDNF levels pre- and post-training. Participants were not required to fast before each blood collection. The blood was allowed to clot for at least 30 min at room temperature and was then centrifuged (Heraeus™ Primo™ R, Thermo Fisher, Waltham, MA) for 10 min at 2200 relative centrifugal force at 20°C. The supernatant was decanted and stored in a -80°C freezer until assayed. Serum concentrations of BDNF were measured using a MILLIPLEX kit (Human Pituitary Bead Panel 2, EMD Millipore, Billerica, MA) according to the manufacturer’s instructions. Samples and BDNF standards were measured in duplicate and results reported in this study were obtained with a sample dilution of 1 part serum to 10 parts sample diluent. The 96-well plate was incubated overnight with agitation on a plate shaker (ThermoMixer® C, Eppendorf, Hamburg, Germany) at 700 rpm and 4°C and the plate was run using MAGPIX® with xPONENT software (Luminex, Austin, TX). The sensitivity as reported in the MILLIPLEX kit literature was 2.45 pg/ml with an intraassay and interassay CV of <10 and <15%, respectively.

### Neuromuscular performance–voluntary activation

Before the cycling trial, participant’s right knee extensor strength was measured on an isokinetic dynamometer (Humac® NORM^TM^, CSMI, MA, USA) whilst seated with their arms folded across their chest and their hips and upper bodies strapped firmly to the seat. In this position, the hip angle was 100° flexion. The right leg was attached to the arm of the dynamometer at a level slightly above the lateral malleolus and the axis of rotation of the arm aligned with the lateral femoral condyle. The dynamometer arm was set so that the knee was 90° from full leg extension. Each participant performed 4 × sub-maximal familiarisation contractions prior to performing 3–6 × 5-s maximal contractions, separated by 1-min recovery periods. Subjects were encouraged verbally to exert the maximal possible force during each contraction. Contractions within a 5% variance were averaged and analysed.

The level of voluntary knee extensor muscle activation was assessed using the twitch interpolation technique [27] before and after the cycling trial. The femoral nerve was stimulated using a constant current peripheral nerve stimulator (Digitimer DS7AH Digitimer, Welwyn Garden City, Hertfordshire, UK) driven by customised software (Labview, version 2010 SP1, National Instruments, Austin, TX, USA). The stimulus was delivered to the skin using self-adhesive electrodes (Verity Medical Ltd, Stockbridge, Hampshire, UK). The cathode was positioned medially on the anterior aspect of the upper thigh ≈ 1 cm below the inguinal fold, while the anode was positioned on the lateral aspect of the upper thigh, between the iliac crest and the greater trochanter. The current was delivered using a single square-wave pulse with a width of 200 μs at 400 V. Initially, the current applied was increased in incremental steps until the resting M wave and evoked twitch torque amplitude plateaus. The current was then increased by a further 10% to ensure supra-maximal activation of the nerve. Six resting twitches were elicited with 10-s rest between stimulations. The level of voluntary knee muscle activation was assessed across three trials with a 1-min rest between each attempt. Participants were instructed to slowly ramp their contraction to attain maximal torque over 2–3 s. Stimulus delivery during contraction was manually triggered by customised software when maximum voluntary contraction (MVC) was attained. Within 5 s following each superimposed MVC, a second stimulus was delivered with the knee extensors at complete rest, determined by the absence of any load placed on the torque strain gauge other than the effect of gravity on the lower limb and the absence of EMG signals from the vastus medialis. The level of voluntary knee extensor muscle activation during each superimposed MVC was calculated using the following equation:

$$Level\;of\;voluntary\;activation\;(\%)=\left[1-(\frac{interpolated\;twitch\;amplitude}{control\;twitch\;amplitude})\right]\times 100$$

where interpolated twitch amplitude was calculated as the maximum torque value produced within 200 ms subsequent to the delivery of the stimulus minus the mean torque value produced during the 50 ms period immediately prior to stimulus delivery and control twitch amplitude is the maximal torque value produced following stimulation whilst the muscle is at complete rest. The level of voluntary knee extensor muscle activation was assessed for all attempts with the single highest value used for further analysis.

### Near-infrared spectroscopy (NIRS)

A continuous-wave NIRS instrument (Oxymon MKIII, Artinis Medical Systems B.V., Zetten, the Netherlands) was used to examine changes in oxygenated ([O_2_Hb]) and deoxygenated ([HHb]) cerebral and muscle tissue haemoglobin concentrations throughout the cycling time trial. For a full description of NIRS method and analysis see [28, 29]. One NIRS probe was placed over the left prefrontal lobe between Fp1 and F3 (international EEG 10–20 system) [30] and adjusted by <5 mm to optimize signal strength [24]. The placement sites were cleaned with an alcohol swab with the inter-optode distance set at 35 mm using a black, plastic spacer affixed to the skin with double-sided self-adhesive disks. Black elastic covering was wrapped over the probes to further secure placement and minimize the intrusion of ambient light. Changes in [O2Hb] and [HHb] were calculated using a modified Beer–Lambert law based on the absorption coefficient of continuous wavelength infrared light (856 and 764 nm) and age-dependent differential path-length factors (range: 5.76–5.85) [24, 31]. NIRS data were recorded at 10 Hz and averaged over a 10 s period [e.g., 2:10–2:20 min for the first lower intensity (L) section and 4:40–4:50 for the first sprint (S)]. The [O_2_Hb] and [HHb] in the present study are reported as changes against a 120 s baseline value collected before the commencement of each session while participants sat quietly on the cycle ergometer with their eyes closed. Oxysoft software was used to apply kernel smoothing with a Gaussian filter, inspect for the presence of movement artifact before exporting raw NIRS data and transferring into excel based on rest and exercise sections. For each of the rest and exercise recordings, average HbO_2_ and HHb concentrations were determined and transferred to GraphPad Prism (Version 6.05, GraphPad Software, Inc., La Jolla, CA) for statistical analysis and graphing.

### Performance time trial, physiological responses and perceived exertion

Power output, heart rate, core and skin temperatures were monitored continuously. Data were averaged over a 20 s period [e.g., 2:05–2:25 min for the first lower intensity (L) section and 4:35–4:55 for the first sprint (S)].

Power output derived from Velotron 3D software (Version 1.0, RacerMate Inc., Seattle, WA) was normalised to lean leg mass and calculated as power output (W)/lean leg mass (kg). Each participant was fitted with a sensor belt (Equivital™ EQ02 LifeMonitor Sensor Belt, Hidalgo, Cambridge, U.K.) to monitor heart rate. Core temperature (T_c_) was monitored via an ingestible telemetric sensor (VitalSense® Core Body Temperature Capsule, Respironics Inc., Murrysville, PA). Sparling, Snow, and Millard-Stafford (1993) reported similar data when comparing ingestion intervals of 3–4 h vs 8–9 h whilst Qi Yin Ng (2008) implemented an ingestion interval of between 6 h 15 min to 7 h 15 min. Thus, our pilot studies indicated that 5 hours before performing the cycling time trial was the optimum interval to ingest the core temperature capsule in the present population. Skin temperature (T_sk_) was measured by iButtons® (Thermodata Viewer, 3.1.19) programmed according to the manufacturer’s instructions and affixed to the skin using Transpore™ tape (3M Health Care, St. Paul, MN) at the chest, arm, thigh and leg (Ramanathan, 1964) immediately before entering the climate chamber. Heart rate and T_c_ signals were transmitted to a sensor electronics module (Equivital™ EQ02 LifeMonitor, Hidalgo, Cambridge, U.K.) and monitored by eqView Professional software (Version 4.1.59.6907). Rating of Perceived Exertion (RPE) was assessed using the Borg 6–20 Scale [32] every 2.5 min. Whole blood lactate (BLa) was assessed from blood taken from the ear lobe with a handheld analyzer (Lactate Pro, Arkray KDK, Kyoto, Japan) immediately after every sprint.

### Exercise training

The intervention followed a mixed method of training. The first session was aerobic, the second session was strength, and the third session was mixed aerobic and strength for a total of 3 sessions per week. At the third session, participants could start with either aerobic or strength training. Aerobic exercises were performed on the cycle ergometer, treadmill or rowing machine and progressed from 15–55 min over the 12 weeks. All bouts of aerobic exercise followed the time trial protocol of 30 s maximal effort sprint for every 4.5 min of lower intensity, and targeted an intensity of > 70% peak heart rate for the lower intensity sections and > 80% peak heart rate for the sprint sections. Resistance exercises targeting the major muscle groups were performed on pulley-weight machines (chest press, seated row, lat pulldown, shoulder press, leg press, leg curl, squats and lunges). Participants progressed from 2 sets of 3 exercises to 4 sets of 8 exercises over the 12 weeks. All sets were performed at loads permitting completion of 10 repetitions, with loads increasing when 12–15 repetitions were completed on the final set. At the first mixed session, 15 min of aerobic activity and 2 sets of 3 strength exercises were performed. At the final mixed session, 27 min aerobic activity and 2 sets of 8 strength exercises were performed. All exercise sessions were fully supervised by qualified personnel and attained 100% compliance.

### Data analysis

All statistical analyses were performed with Prism 6 software (Version 6.05, GraphPad Software, Inc., La Jolla, CA) for Windows. A priori power analysis for a within-factors repeated-measures ANOVA was performed using G*Power v3.1 software. The input parameters were the desired effect size (0.5), Type I error probability of 0.05, desired statistical power of 0.80, number of groups (2), and number of measurements (10). These inputs yielded an actual power 1-ß = 0.95, and a total sample size of n = 6. Given these outcomes we employed a sample size of n = 8 for the experiment. A Shapiro-Wilk test for normality was conducted and all data met the assumptions of linear statistics (*P* > 0.05). The effect of training on measures obtained before and after the cycling time trials were identified through a paired *t*-test. Between and within group differences in measures obtained during the time trial were identified through a repeated measures two-way ANOVA. The source of significance was located using uncorrected Fisher’s LSD test.

Data are presented as mean ± SD unless otherwise indicated with significance set at *P* < 0.05. Effect sizes were calculated by dividing the difference in means by average standard deviation, and magnitudes were assessed using the following criteria: ≤ 0.19 = trivial, 0.20–0.49 = small, 0.50–0.79 = moderate, and > 0.80 = large [33].

## Results

### Participant characteristics

The participant characteristics are given in Table 1. At post-training total body mass decreased by 0.6±1.8 kg (Table 1). Whole body fat percentage decreased by 1.5±2.2% (*P* = 0.048) whilst total lean body mass increased by 1.4±2.1 kg (*P* = 0.047).

**Table 1:** Mean ± SD (range) of age and physical characteristics for eight male participants.

	Pre-training	Post-training	Effect size, *d*
*Age (years)*	58.6±4.2 (52.5–63.7)		
*Height (cm)*	176.7±7.0 (168.2–189.6)		
*Mass (kg)*	87.4±8.9 (76.5–97.0)	86.8±8.0 (76.4–96.7)	-0.07
*DXA*			
Total fat (%)	30.3±2.8 (27–36)	28.8±1.5 (27–31)	-0.70 *
Total lean mass (kg)	59.8±5.6 (51.3–67.3)	61.3±6.1 (53.1–68.3)	0.25^#^

Significance post-training indicated by

* *P* = 0.048

^#^*P* = 0.047

DXA = dual energy X-ray absorptiometry

Effect size: ≤ 0.19 = trivial, 0.20–0.49 = small, 0.50–0.79 = moderate, and > 0.80 = large [33]

### BDNF

Post training serum BDNF decreased from resting values by 3.06±2.79 ng/ml (*P* = 0.017), representing a 31% change post-training (Table 2).

**Table 2:** Mean±SD (range) of BDNF, time trial measures and evoked twitch properties.

Variable	Pre-training	Post-training	Effect size, *d*
*BDNF (ng/ml)*	10.02±4.63 1.35–18.35)	6.96±3.56 (1.53–13.21)	-0.75 *
*Time trial*			
Distance (km)	11.40±1.07 (10.25–13.58)	11.26±1.06 (9.42–13.01)	-0.14
Normalised peak power (W/kg)	45.9±9.6 (36.0–67.0)	48.9±5.9 (39.8–57.2)	0.39
Peak heart rate (beats/min)	161±16 (139–180)	154±15 (129–173)	-0.44 *
*Evoked resting twitch* ^a^			
Peak force (N)	30.5±10.1 (18.2–46.7)	34.3±12.2 (21.9–56.2)	0.34
Rate of force development (N/s)	355.2±130.3 (203.8–569.1)	395.1±142.5 (246.9–660.6)	0.29
Time to peak force (ms)	86.5±3.6 (82.0–90.0)	86.6±5.4 (79.0–95.5)	0.02
Half relaxation time (ms)	46.3±17.9 (31.0–81.0)	53.0±22.2 (28.5–84.0)	0.34
Contraction duration (ms)	132.8±19.3 (114.5–170.0)	139.6±21.5 (123.0–172.5)	0.33

^a^*N* = 6

Significance post-training indicated by

*P = 0.024

BDNF = brain-derived neurotrophic factor

Effect size: ≤ 0.19 = trivial, 0.20–0.49 = small, 0.50–0.79 = moderate, and > 0.80 = large [33]

### Neuromuscular performance and muscle quality

Due to technical issues, only the results from 6 participants were analysed and presented. The resting evoked twitch properties remained unchanged with training (Table 2). Before the time trial, pre-training the voluntary activation was 89.9±10.6% and 86.1±4.5% post-training. This change was not significant with a moderate effect (-0.51). following the time trial, the pre-training voluntary activation was 87.6±6.4% and 85.5±2.2% post-training. This change was not significant with a small effect (-0.48). The time trial did not have an effect on voluntary activation pre- or post-training.

### Near-infrared spectroscopy (NIRS)

**Fig 3:**
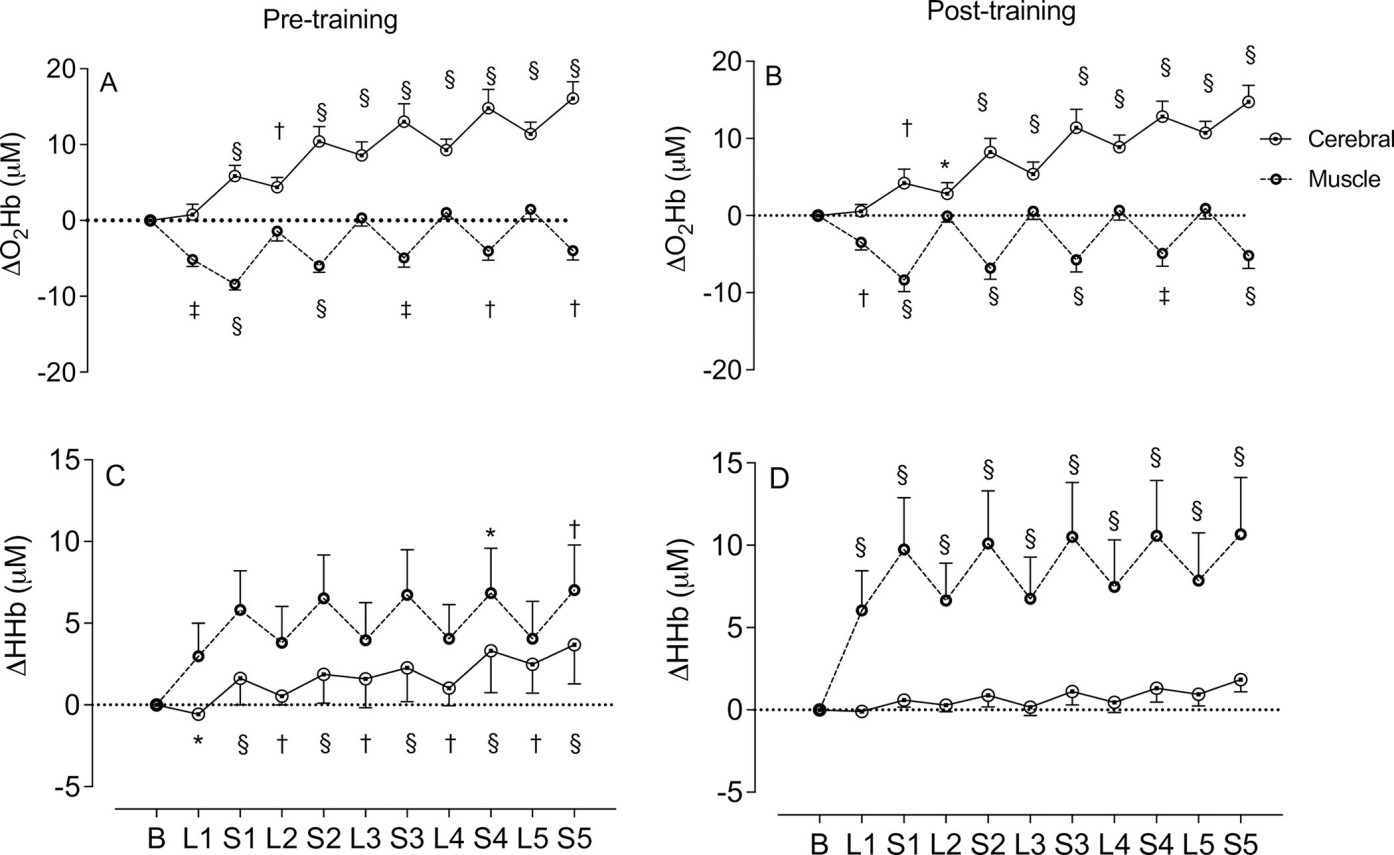
Changes from resting values in cerebral and muscle tissue oxygenation during the cycling time trial. *A*: Changes in O_2_Hb pre-training. *B* Changes in O_2_Hb post-training. *C*: Changes in HHb pre-training. *D*: Changes in HHb post-training. Significance from baseline indicated by * *P* < 0.02, ^†^ *P* < 0.01, ^‡^ *P* < 0.001 and ^§^ *P* < 0.0001. [O_2_Hb] = concentration of oxyhaemoglobin; [HHb] = concentration of deoxyhaemoglobin; L = lower intensity; S = sprint. Values are presented as mean ± SEM.

There was an interaction effect for O_2_Hb for both pre- and post-training (*P* = 0.0001). Cerebral Δ[O_2_Hb] increased from S1 to the end of the cycling time trial pre- (Fig 3A) and post-training (Fig 3B) whilst changes in the muscle Δ[O_2_Hb] occurred primarily during the sprints (Fig 2A and 2B). Pre-training cerebral Δ[HHb] increased from baseline throughout the test while muscle Δ[HHb] increased for the last two sprint intervals (Fig 3C). Post-training, there was an increase in muscle Δ[HHb] throughout the cycling time trial while no changes were observed in the cerebral Δ[HHb] (Fig 3D).

### Performance time trial, physiological responses and RPE

The decrease in total distance of 0.14±0.72 km and increase in normalised peak power of 3.0±9.3 W/kg were not significant after training (Table 2). Peak heart rate was significantly lower post-training (*P* = 0.024).

**Fig 4:**
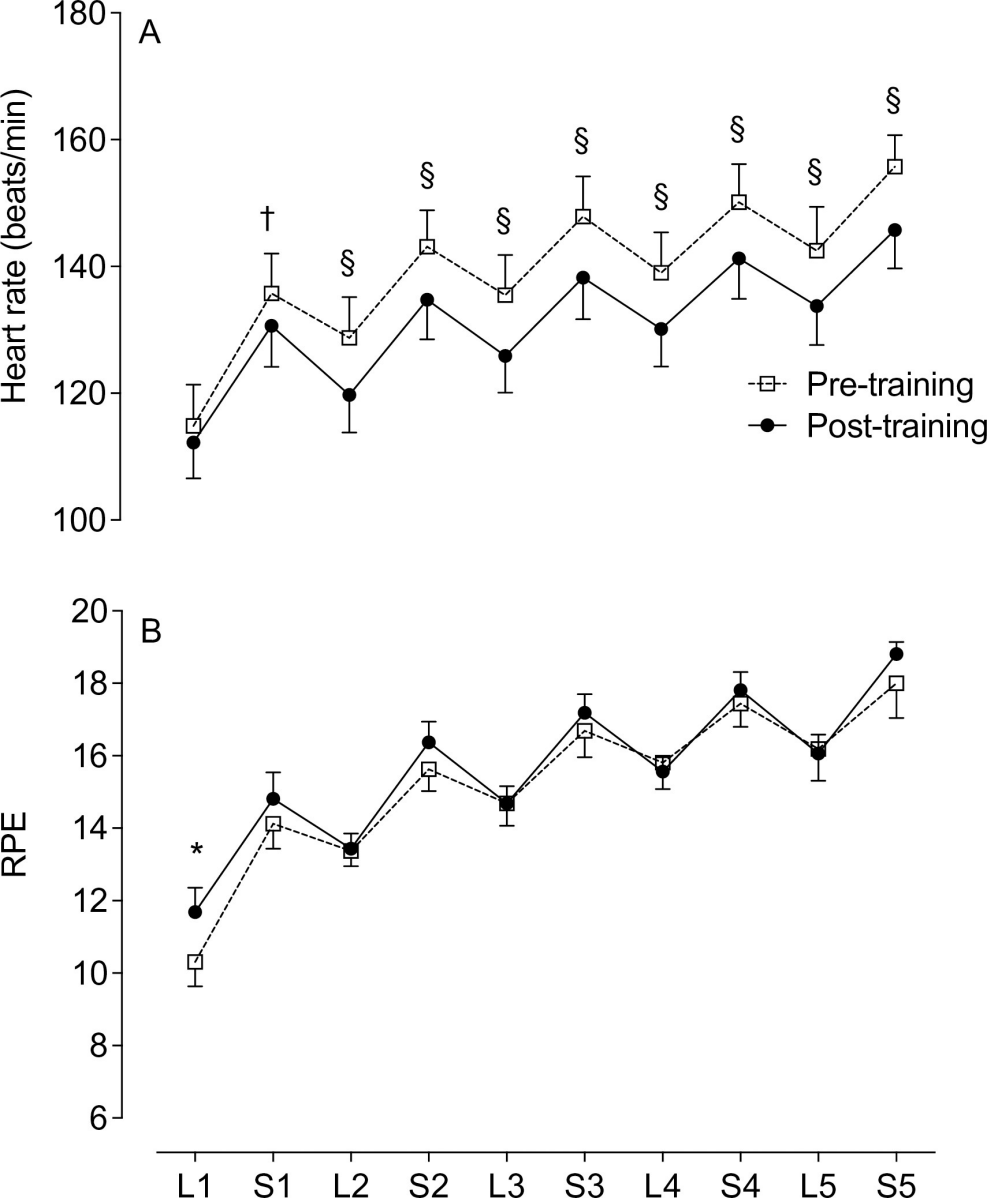
Heart rate (*A*) and RPE (*B*) during the cycling time trial Significance post-training indicated by * *P* < 0.05, ^†^ *P* < 0.01, ^§^ *P* < 0.0001. L = lower intensity; S = sprint. Data presented as mean ± SEM.

Heart rate was significantly lower post-training at L2–5 (*P* = 0.0001), S1 (*P* = 0.002) and S2–5 (*P* < 0.0001) (Fig 4A). Pre-training, the mean heart rate at the start of exercise was 115 beats/min and 156 beats/min at S5. Post-training, the mean heart rate at the start of exercise was 112 beats/min and 146 beats/min at S5. There was no interaction effect for heart rate during the lower intensity and sprint sections.

RPE during the time trial rose from 10.3±1.9 to 16.2±2.5 (pre-training) and from 11.7±1.9 to 16.2±2.5 (post-training) during the lower intensity sections, with a significant higher rating only at L1 post-training (*P* = 0.016; Fig 3B). No differences were observed in the RPE during the sprint sections, with a rise from 14.1±2.0 to 18.0±2.7 (pre-training) and from 14.8±2.1 to 18.8±0.9 (post-training; Fig 4B). For both pre- and post-training, the RPE at the end of the 25 min TT corresponds to ‘Very hard’ and ‘Extremely hard’. There was no interaction effect for RPE during the lower intensity and sprint sections.

Overall, BLa was lower across all sprints post-training with significance at S1 (*P* = 0.018) and 4 (*P* = 0.012). Pre-training, the mean BLa after S1 was 6.0±1.8 and 9.5±1.5 mmol/l after S5. Post-training, the mean BLa after S1 was 5.2±1.7 and 9.1±1.9 mmol/l after S5.

**Fig 5:**
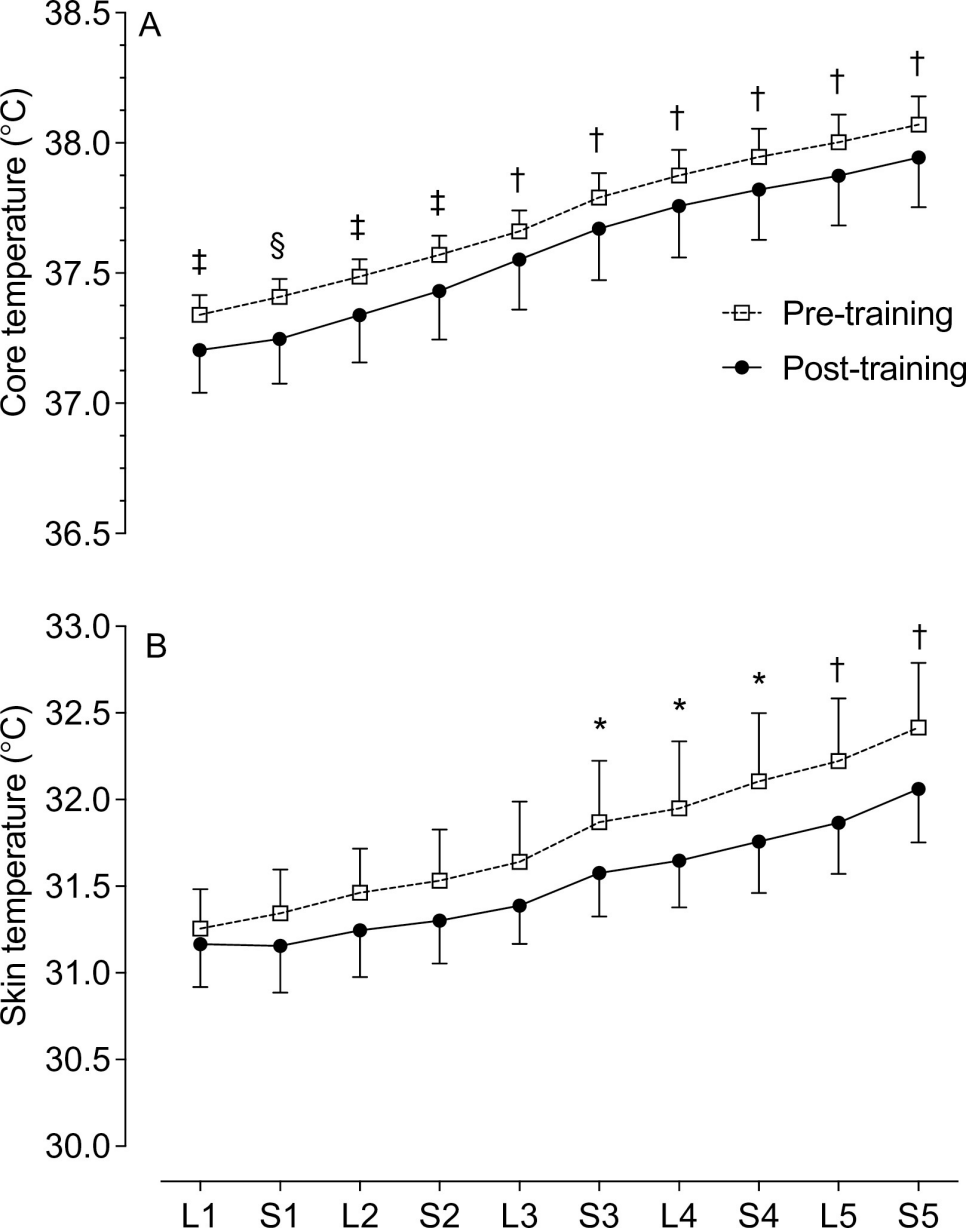
Thermoregulatory responses during the cycling time trial. *A*: Core temperature. *B*: Skin temperature. Significance post-training indicated by * *P* < 0.02, ^†^ *P* < 0.01, ^‡^ *P* < 0.001, ^§^ *P* < 0.0001. L = lower intensity; S = sprint. Data presented as mean ± SEM.

The T_c_ was lower throughout the entire 25 min of cycling post-training compared to pre-training (*P* = 0.0001). The T_c_ increased from 37.34±0.21°C at the start of cycling to 38.07±0.31°C (*P* = 0.01) by the end of 25 min pre-training and increased from 37.20±0.46 to 37.94±0.54°C (*P* = 0.01) post-training (Fig 5A). There was no interaction effect for T_c_ during the lower (*P* = 0.945) intensity and sprint (*P* = 0.926) sections.

The T_sk_ increased from 31.26±0.64°C at the start of cycling to 32.42±1.05°C (*P* = 0.01) at the end of 25 min pre-training and increased from 31.17±0.70 to 32.06±0.87°C (*P* = 0.01) post-training. The T_sk_ was lower post-training during the last 10 min of cycling (*P* = 0.009) (Fig 5B). There was no interaction effect for T_sk_ during the lower (*P* = 0.65) intensity and sprint (*P* = 0.85) sections.

**Fig 6:**
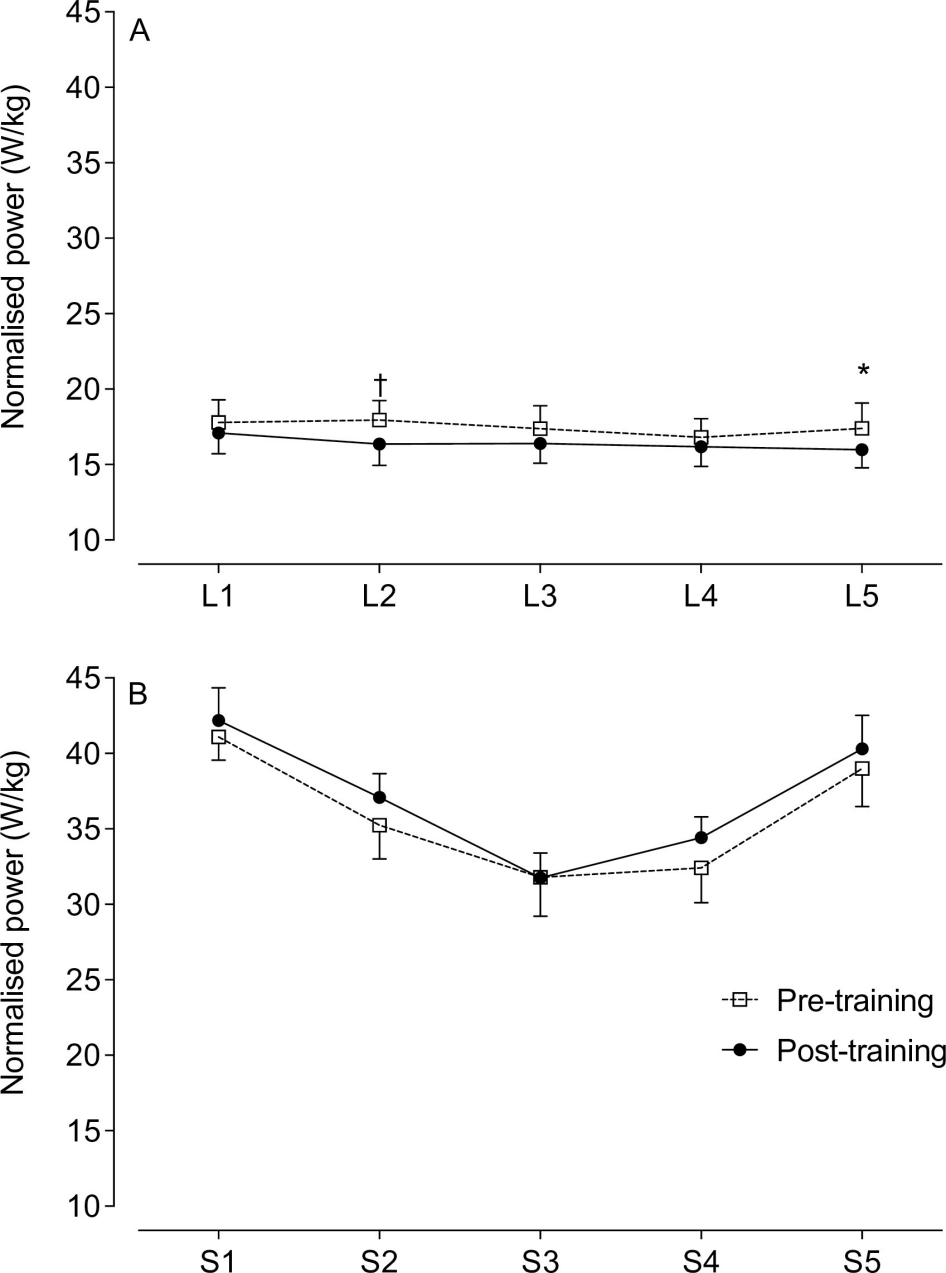
Normalised power output during the lower intensity (*A*) and sprint sections (*B*) of the cycling time trial. Power output normalised to lean leg mass. Significance post-training indicated by * *P* < 0.01, ^†^ *P* < 0.005. L = lower intensity; S = sprint. Data presented as mean ± SEM.

Normalised power is shown in Fig 6. During the lower intensity sections, the mean normalised power output ranged from 16.8–18.0 W/kg pre-training and 16.0–17.1 W/kg post-training, with a significantly lower power output post-training at L2 (*P* = 0.004) and L5 (*P* = 0.011); Fig 6A). During the sprint sections, there were no significant changes in normalised power output post-training (pre-training = 31.8–41.1W/kg; post-training = 31.8–42.2 W/kg; Fig 6B). For both time trials, power output commenced at ≈ 42 W/kg and significantly decreased to ≈ 32 W/kg by the S3 and was then restored in S5 to ≈ 40 W/kg. There was no interaction effect for normalised power output during the lower intensity and sprint sections. Overall, the power output increased during the sprints and decreased during the lower intensity sections, with corresponding changes in heart rate.

## Discussion

Our findings show that exercise training changed the physiological responses even though exercise performance was not improved in a self-paced cycling trial. The favourable outcomes in body composition are in agreement with the literature [34]. However, the lack of improvement in performance is contradictory to other research where older adults improved with aerobic exercise, resistance exercise, high-velocity resistance exercise and a combination of aerobic and resistance exercise training [35, 36]. It is clear that concurrent aerobic and resistance exercise training in the present study did not provide specific experience to improve performance in the cycling time trial. That is, specific training which allows individuals to experience the pacing during a time trial is more likely to be required to improve pacing strategies in previously untrained older males. This is a key finding as it supports the contention that pacing is a cognitive process likely to be dependent on prior experience and the associated physiological changes and not solely on improved physiological capacity.

Similar to the lack of optimal pacing strategies in schoolchildren [11], our sample of adult males were unable to develop an improved pacing strategy after 12 weeks of combined aerobic and resistance exercise training. These findings strengthen the view that pacing is a complex phenomenon. Although our data does not provide a direct explanation as to why performance in the cycling time trial did not occur in tandem with cardiovascular improvements such as a reduction in heart rate 12 weeks post-training, it does shed light on what other elements of exercise training may be required to improve performance beyond the improved level of fitness in older men.

Despite the fact that physiological responses did not reflect any improvements in performance, the participants were able to sustain the same level of performance with less physiological strain. Heart rate, peak heart rate, T_c_ and T_sk_ obtained during the post training time trial were lower, whilst blood lactate and RPE at the end of S5 reached values indicating exercise was performed at a high intensity during both pre- and post-training trials. This suggests positive health outcomes for previously sedentary individuals whose exercise goals are not necessarily to improve performance but to maximise physiological function and improve their health status.

Based on our findings, we reject our original hypothesis that chronic exercise training would result in increases in BDNF values leading to improved pacing during self-regulated cycling. However, it is pertinent to note that in the present study, 12 weeks of mixed aerobic and resistance exercise training was sufficient to elicit a decrease in serum BDNF, the opposite of our original hypothesis. Studies report equivocal effects of exercise on BDNF levels. Seifert et al. [37] reported increased concentrations of resting jugular venous BDNF in younger men after 3 months of aerobic exercise training, indicating improved brain health with enhanced release of BDNF from the brain with exercise training. In contrast, lower basal serum BDNF concentrations have been observed in highly trained compared to less trained healthy men [38, 39]. In active versus sedentary females, serum BDNF values immediately post maximal exercise increased, but within 30 min, this value had decreased below baseline and continued to decrease at 60 min post exercise to be ~20% below baseline [40]. Similarly, others have reported that following 2 and 6 months of training (either endurance or high intensity), BDNF levels significantly decreased at 10 and 60 min post exercise in trained cyclists [41]. More recently, in a study of marathon runners (23% female, mean age 43 years) over a period of 6 months, measures of serum BDNF after the marathon race at 24 to 72 h and at 3 months follow-ups, values were significantly lower at 72 h compared to baseline which was more pronounced in the males [42]. The reasons for the reported decrease in BDNF values post exercise have been largely attributed to the possible role of BDNF utilization is the repair of exercise-induced muscle damage, although these findings are based mainly on rodent studies [43]. These studies extrapolated the potential function of BDNF following its administration which suppressed the release of creatine kinase and prostaglandin E2, both of which are indicators of muscle cell damage and that delayed regeneration of muscle fibres following injury was observed in muscle-specific BDNF knockout mice.

Although, we are unable to make direct comparisons for our results with respect to decreased BDNF values following post training due to the differences in study design, we measured the post-training value of BDNF within approximately 20 min of the last training session, before participants progressed to the post-training time trial. Given that decreases in BDNF have been found in trained individuals from 10 min [41] and up to 72 h [42] post exercise, we speculate that our BDNF values were likely influenced by the last training session. As such, our results would be in line with these previous findings since our participants were trained for 12 weeks and the training sessions will have produced some skeletal muscle cell damage.

Notwithstanding that we did not assess cognitive function *per se*, participants were all healthy and well-functioning so that degenerative cognitive changes were not likely to be observed over 12 weeks. Thus, changes in neurotrophin levels were observed in the absence of performance improvements. These findings support a previous hypothesis that lower levels of peripheral BDNF in trained individuals is indicative of neuroprotective upregulation of BDNF into the central nervous system, resulting in less circulating peripheral BDNF [39]. Notably, the incidence of dementia has been demonstrated to be lower in persons 65 years and older who exercised three or more times per week than those who exercise fewer than three times per week [44] and a higher level of total daily physical activity is associated with a reduced risk of Alzheimer disease [45].

In the assessment of central fatigue, peripheral electrical stimulation is delivered to the motor axons innervating the muscle during voluntary muscle contraction to measure the level of voluntary activation [46]. Peripheral sources of fatigue include neuromuscular propagation and contractile properties which can be indirectly measured by the amplitude of the compound muscle action potential (M-wave) in response to a peripheral electrical stimulation and the relaxation time. Studies examining the age-related effect on neuromuscular fatigue are equivocal largely owing to inconsistencies in task and methodology and differences in sex, physical activity status, lack of motivation and practice. The lack of significant differences in the voluntary activation in the present study may indicate no changes in central activation although the moderate effect size indicates the decrease may be individually meaningful. Furthermore, no observable difference in the resting evoked twitch indicates the lack of changes to the peripheral contractile properties with exercise training [6].

Measurement of cerebral and muscle tissue haemodynamics indicate central and peripheral changes in [HHb]. Pre-training, cerebral [HHb] was significantly different from baseline values whilst no differences were observed post-training, indicating systemic hypoxemia pre-training. [47] In the muscle, [HHb] was significantly different from baseline values from L1 to the end of the cycling time trial post-training, whilst changes were observed only in S4 and S5 pre-training, indicating increased oxygen delivery and utilisation post-training [22]. These improvements in blood flow and oxygen extraction occurred without a concomitant improvement in cycling time trial performance and consistent with the lower values in heart rate and thermoregulatory responses. These findings also indicate that merely improving physiological function and overall fitness will not necessarily lead to improved specific performance or an altered pacing strategy in previously untrained healthy older men.

Although traditional submaximal exercise performance tests show poor reproducibility [48], self-paced performance tests are thought to provide ecological validity and improved reproducibility [49]. However, more recent literature suggests that there are additional considerations in interpreting findings from self-paced exercise protocols. For instance, pacing is employed so that exercise can be completed without exhaustion and the maintenance of homeostasis [50]. Our protocol was interspersed with bouts of high intensity efforts which might have limited our participants in obtaining the usually more pronounced end-spurt [51]. Although we observed a reduced HR over the time trial (Fig 3A) following the 12 weeks of concurrent training, the RPE was similar for pre and post training (Fig 3B), indicating that a higher pacing strategy was not adopted as shown by the normalised power output (Fig 5). Since RPE is generally considered to be the regulator of muscular work, it is possible that our protocol with interspersed high intensity efforts provided additional afferent feedback which may have also attenuated the potential neural drive resulting in similar pre and post training power output [52].

## Conclusions

In conclusion, the exercise training program in the present study did not elicit specific improvements in self-paced cycling performance, although it enabled older men to perform at similar levels with apparent attenuated physiological strain. Twelve weeks of concurrent aerobic and resistance training reduced BDNF values possibly due to a neuroprotective upregulation into the CNS [39]. We did not observe improvement in self-regulated cycling exercise, but we suggest that specific training in conjunction with neuroplasticity rather than general training is likely to be a stronger stimulus for altered pacing and performance improvements. However, this hypothesis requires further examination. Finally, although changes in neuromuscular properties were not observed, the positive outcomes of exercise training on body composition have implications for previously sedentary older men to begin exercising and gain from the multitude of physical benefits, which may also confer a neuroprotective effect as evidenced by the altered circulating BDNF.

## Supporting information

S1 Checklist CONSORT 2010 checklist of information to include when reporting a randomised trial*. (DOC)

S1 File Participant information: Details of information provided to participant for the purpose of obtaining written informed consent. (PDF)

## References

[1] Wei JY (1992) Age and the cardiovascular system. N Eng J Med 327: 1735–1739. doi: 10.1056/NEJM199212103272408 1304738

[2] Timothy JD (2003) Physiology of aging invited review: Aging and sarcopenia. J Appl Physiol 95: 1717–1727. 12970377 10.1152/japplphysiol.00347.2003

[3] Cotman CW, Berchtold NC (2002) Exercise: a behavioral intervention to enhance brain health and plasticity. Tren Neurosc 25: 295–301. doi: 10.1016/s0166-2236(02)02143-4 12086747

[4] Seals DR (2014) Edward F. Adolph Distinguished Lecture: The remarkable anti-aging effects of aerobic exercise on systemic arteries. J Appl Physiol 117: 425–439. doi: 10.1152/japplphysiol.00362.2014 24855137 PMC4157159

[5] Klitgaard H, Mantoni M, Schiaffino S, Ausoni S, Gorza L, Laurent‐Winter C et al. (1990) Function, morphology and protein expression of ageing skeletal muscle: a cross‐sectional study of elderly men with different training backgrounds. Acta Physiol Scand 140: 41–54. doi: 10.1111/j.1748-1716.1990.tb08974.x 2275404

[6] Allman BL, Rice CL (2002) Neuromuscular fatigue and aging: Central and peripheral factors. Muscle Nerve 25: 785–796. doi: 10.1002/mus.10116 12115966

[7] Lynch NA, Metter EJ, Lindle RS, Fozard JL, Tobin JD, Roy TA et al. (1999) Muscle quality. I. Age-associated differences between arm and leg muscle groups. J Appl Physiol 86: 188–194. doi: 10.1152/jappl.1999.86.1.188 9887130

[8] Salthouse TA (2009) When does age-related cognitive decline begin. Neurobiol Aging 30: 507–514. doi: 10.1016/j.neurobiolaging.2008.09.023 19231028 PMC2683339

[9] Lex H, Essig K, Knoblauch A, Schack T (2015) Cognitive representations and cognitive processing of team-specific tactics in soccer. PloS one 10: e0118219. doi: 10.1371/journal.pone.0118219 25714486 PMC4340951

[10] St Clair Gibson A, Lambert EV, Rauch LH, Tucker R, Baden DA, Foster C et al. (2006) The role of information processing between the brain and peripheral physiological systems in pacing and perception of effort. Sports Med 36: 705–722. doi: 10.2165/00007256-200636080-00006 16869711

[11] Micklewright D, Angus C, Suddaby J, St Clair Gibson A, Sandercock G, Chinnasamy C (2012) Pacing strategy in schoolchildren differs with age and cognitive development. Med Sci Sports Exerc 44: 362–369. doi: 10.1249/MSS.0b013e31822cc9ec 21796049

[12] Micklewright D, Papadopoulou E, Swart J, Noakes T (2010) Previous experience influences pacing during 20 km time trial cycling. Br J Sports Med 44: 952–960. doi: 10.1136/bjsm.2009.057315 19364755

[13] Chia E, Cannon J, Marino FE (2015) The effects of acute versus chronic training status on pacing strategies of older men in a hot, humid environment. J Therm Biol 53: 125–134. doi: 10.1016/j.jtherbio.2015.10.001 26590465

[14] Van Biesen D, Hettinga FJ, McCulloch K, Vanlandewijck Y (2016) Pacing profiles in competitive track races: regulation of exercise intensity is related to cognitive ability. Front Physiol 7: 624. doi: 10.3389/fphys.2016.00624 28066258 PMC5167700

[15] Lewin GR, Barde Y-A (1996) Physiology of the neurotrophins. Ann Rev Neurosci 19: 289–317. doi: 10.1146/annurev.ne.19.030196.001445 8833445

[16] McAllister AK, Katz LC, Lo DC (1999) Neurotrophins and synaptic plasticity. Ann Rev Neurosci 22: 295–318. doi: 10.1146/annurev.neuro.22.1.295 10202541

[17] Vaynman S, Gomez‐Pinilla F (2006) Revenge of the “sit”: how lifestyle impacts neuronal and cognitive health through molecular systems that interface energy metabolism with neuronal plasticity. J Neurosci Res 84: 699–715. doi: 10.1002/jnr.20979 16862541

[18] Kang JI, Namkoong K, Ha RY, Jhung K, Kim YT, Kim SJ (2010) Influence of BDNF and COMT polymorphisms on emotional decision making. Neuropharm 58: 1109–1113. doi: 10.1016/j.neuropharm.2010.02.001 20153759

[19] Huang T, Larsen KT, Ried-Larsen M, Møller NC, Andersen LB (2014) The effects of physical activity and exercise on brain-derived neurotrophic factor in healthy humans: A review. Scand J Med Sci Sports 24: 1–10. doi: 10.1111/sms.12069 23600729

[20] Hirth C, Obrig H, Valdueza J, Dirnagl U, Villringer A (1997) Simultaneous assessment of cerebral oxygenation and hemodynamics during a motor task. editors. Springer. pp. 461–469. 10.1007/978-1-4615-5865-1_59 9269463

[21] Boushel R, Langberg H, Olesen J, Gonzales-Alonzo J, Bülow J, Kjær M (2001) Monitoring tissue oxygen availability with near infrared spectroscopy (NIRS) in health and disease. Scan J Med Sci Sports 11: 213–222. doi: 10.1034/j.1600-0838.2001.110404.x 11476426

[22] DeLorey DS, Kowalchuk JM, Paterson DH (2004) Effect of age on O2 uptake kinetics and the adaptation of muscle deoxygenation at the onset of moderate-intensity cycling exercise. J Appl Physiol 97: 165–172. doi: 10.1152/japplphysiol.01179.2003 15003999

[23] Obrig H, Hirth CHRISTINA, Junge-Hulsing JG, Doge C, Wolf T, Dirnagl U et al. (1996) Cerebral oxygenation changes in response to motor stimulation. J Appl Physiol 81: 1174–1183. doi: 10.1152/jappl.1996.81.3.1174 8889751

[24] Billaut F, Davis JM, Smith KJ, Marino FE, Noakes TD (2010) Cerebral oxygenation decreases but does not impair performance during self-paced, strenuous exercise. Acta Physiol 198: 477–486. 10.1111/j.1748-1716.2009.02058.x 19912150

[25] Granacher U, Gruber M, Gollhofer A (2009) Resistance Training and Neuromuscular Performance in Seniors. Int J Sports Med 30: 652–657. doi: 10.1055/s-0029-1224178 19569007

[26] De Pauw K, Roelands B, Cheung SS, De Geus B, Rietjens G, Meeusen R (2013) Guidelines to classify subject groups in sport-science research. Int J Sports Physiol Perf 8: 111–122. doi: 10.1123/ijspp.8.2.111 23428482

[27] Shield A, Zhou S (2004) Assessing voluntary muscle activation with the twitch interpolation technique. Sports Med 34: 253–267. doi: 10.2165/00007256-200434040-00005 15049717

[28] Immink MA, Pointon M, Wright DL, Marino FE (2021) Prefrontal cortex activation during motor sequence learning under interleaved and repetitive practice: A two-channel near-infrared spectroscopy study. Front Hum Neuro 15: Article 644968. 10.3389/fnhum.2021.644968 PMC8160091 34054448

[29] Minett GM, Duffield R, Billaut F, Cannon J, Portus MR, Marino FE (2014) Cold-water immersion decreases cerebral oxygenation but improves recovery after intermittent-sprint exercise in the heat. Scand J Med Sci Sports 24: 656–666. doi: 10.1111/sms.12060 23458430

[30] Perrey S (2008) Non-invasive NIR spectroscopy of human brain function during exercise. Methods 45: 289–299. doi: 10.1016/j.ymeth.2008.04.005 18539160

[31] Duncan A, Meek JH, Clemence M, Elwell CE, Fallon P, Tyszczuk L et al. (1996) Measurement of cranial optical path length as a function of age using phase resolved near infrared spectroscopy. Ped Res 39: 889–894. doi: 10.1203/00006450-199605000-00025 8726247

[32] Borg GAV (1982) Psychophysical bases of perceived exertion. Med Sci Sports Exerc 14: 377–381. 7154893

[33] Cohen J (1988) Statistical power analysis for the behavioral sciences. Hillsdale (NJ): Lawrence Erlbaum Associates. 18 p.

[34] Chodzko-Zajko WJ, Proctor DN, Singh MAF, Minson CT, Nigg CR, Salem GJ et al. (2009) Exercise and physical activity for older adults. Med Sci Sports Exerc 41: 1510–1530. 19516148 10.1249/MSS.0b013e3181a0c95c

[35] Frontera WR, Bigard X (2002) The benefits of strength training in the elderly. Sci Sports 17: 109–116.

[36] Van Roie E, Delecluse C, Coudyzer W, Boonen S, Bautmans I (2013) Strength training at high versus low external resistance in older adults: effects on muscle volume, muscle strength, and force–velocity characteristics. Exp Gerentol 48: 1351–1361. doi: 10.1016/j.exger.2013.08.010 23999311

[37] Seifert T, Brassard P, Wissenberg M, Rasmussen P, Nordby P, Stallknecht B et al. (2010) Endurance training enhances BDNF release from the human brain. Am J Physiol Reg Int Comp Physiol 298: R372–R377. doi: 10.1152/ajpregu.00525.2009 19923361

[38] Chan KL, Tong KY, Yip SP (2008) Relationship of serum brain-derived neurotrophic factor (BDNF) and health-related lifestyle in healthy human subjects. Neuro Lett 447: 124–128. doi: 10.1016/j.neulet.2008.10.013 18852019

[39] Currie J, Ramsbottom R, Ludlow H, Nevill A, Gilder M (2009) Cardio-respiratory fitness, habitual physical activity and serum brain derived neurotrophic factor (BDNF) in men and women. Neurosc Lett 451: 152–155. doi: 10.1016/j.neulet.2008.12.043 19133315

[40] Nofuji Y, Suwa M, Sasaki H, Ichimiya A, Nishichi R, Kumagai S (2012) Different circulating brain-derived neurotrophic factor responses to acute exercise between physically active and sedentary subjects. J Sports Sci Med 11: 83. 24137066 PMC3737858

[41] Hebisz P, Hebisz R, Murawska-Ciałowicz E, Zatoń M (2019) Changes in exercise capacity and serum BDNF following long-term sprint interval training in well-trained cyclists. Changes in exercise capacity and serum BDNF following long-term sprint interval training in well-trained cyclists 44: 499–506. doi: 10.1139/apnm-2018-0427 30286300

[42] Roeh A, Holdenrieder S, Schoenfeld J, Haeckert J, Halle M, Falkai P et al. (2021) Decreased serum brain-derived neurotrophic factor concentrations 72 hours following marathon running. Front Physiol 1001. doi: 10.3389/fphys.2021.668454 34335291 PMC8320388

[43] Clow C, Jasmin BJ (2010) Brain-derived neurotrophic factor regulates satellite cell differentiation and skeltal muscle regeneration. Mol Biol Cell 21: 2182–2190. doi: 10.1091/mbc.e10-02-0154 20427568 PMC2893983

[44] Larson EB, Wang LI, Bowen JD, McCormick WC, Teri L, Crane P et al. (2006) Exercise is associated with reduced risk for incident dementia among persons 65 years of age and older. Ann Int Med 144: 73–81. doi: 10.7326/0003-4819-144-2-200601170-00004 16418406

[45] Buchman AS, Boyle PA, Yu L, Shah RC, Wilson RS, Bennett DA (2012) Total daily physical activity and the risk of AD and cognitive decline in older adults. Neurol 78: 1323–1329. doi: 10.1212/WNL.0b013e3182535d35 22517108 PMC3335448

[46] Gandevia S (2001) Spinal and supraspinal factors in human muscle fatigue. Spinal and supraspinal factors in human muscle fatigue 81: 1726–1789. doi: 10.1152/physrev.2001.81.4.1725 11581501

[47] Nybo L, Rasmussen P (2007) Inadequate cerebral oxygen delivery and central fatigue during strenuous exercise. Exerc Sports Sci Rev 35: 110–118. doi: 10.1097/jes.0b013e3180a031ec 17620929

[48] McLellan TM, Cheung SS, Jacobs I (1995) Variability of time to exhaustion during submaximal exercise. Can J Appl Physiol 20: 39–51. doi: 10.1139/h95-003 7742769

[49] Marino FE, Kay D, Cannon J, Serwach N, Hilder M (2002) A roproducible and variable intensity cycling performance protocol for warm conditions. J Sci Med Sport 5: 95–107. 12188090 10.1016/s1440-2440(02)80030-5

[50] Smits BL, Pepping GJ, Hettinga FJ (2014) Pacing and decision making in sport and exercise: the roles of perception and action in the regulation of exercise intensity. Sports Med 44: 763–775. doi: 10.1007/s40279-014-0163-0 24706362

[51] Lima-Silva AE, Correia-Oliveira CR, Tenorio L, Melo AA, Bertuzzi R, Bishop D (2013) Prior exercise reduces fast-start duration and end-spurt magnitude during cycling time-trial. Int J Sports Med 34: 736–741. doi: 10.1055/s-0032-1331258 23325716

[52] Renfree A, Martin L, Micklewright D, St Clair Gibson A (2014) Application of decision-making theory to the regulation of muscular work rate during self-paced competitive endurance activity. Sports Med 44: 147–158. doi: 10.1007/s40279-013-0107-0 24113898

