# Supplementary material for: Serum brain-derived neurotrophic factor (BDNF) and self-paced time-trial performance in older untrained men
Source: PLoS One. 2023 Jul 3;18(7):e0285628. doi: 10.1371/journal.pone.0285628 (PMC10317232; doi:10.1371/journal.pone.0285628)
Supplement: S1 File — (PDF) [file pone.0285628.s002.pdf]

[redacted]

[redacted]

[redacted]

[redacted]

## Information Sheet – Training Study

### 1 Title of Research Proposal

The Effects of Age and Training on Fatigue, Pacing, and Thermoregulation

### 2 Investigators & Contact Details


Ms Ee Von Chia
PhD Candidate
School of Human Movement Studies
Charles Sturt University
Tel: +61 408 721 752
Email: echia@csu.edu.au

Prof Frank Marino
Principle Supervisor
School of Human Movement Studies
Charles Sturt University
Tel: +612 6338 4268
Email: fmarino@csu.edu.au

Dr Jack Cannon
Co-Supervisor
School of Human Movement Studies
Charles Sturt University
Tel: +612 6338 4334
Email: jcannon@csu.edu.au

Dr Eric Drinkwater
Co-Supervisor
School of Human Movement Studies
Charles Sturt University
Tel: +612 6338 6116
Email: edrinkwater@csu.edu.au


### 3 Purpose of the Research

The primary purpose of the proposed research is to examine the response of older adults to a bout of exercise in ambient and hot environments, their choice of pacing, and profile their recovery over a 24-hour period. This research also aims to examine the effects of a training programme on sedentary older adults. The findings from this research may be used to develop strategies to optimise performance and investigate the reasons for reduced performance in older adults.

## 4 Procedures of the Research

Ten untrained healthy males aged 50–65 years will be recruited for this study. You will be required to attend a baseline session where testing will be carried out to measure your height, weight, and body composition. Aerobic fitness will be determined on a cycle ergometer. After a rest period of at least a week, you will return to the laboratory for a familiarisation session. During this session, you will perform maximal voluntary contractions of the quadriceps and elbow flexors with magnetic stimulation at the cerebral and muscle tissue. You will also be familiarised with the entire cycling trial which includes 4 cycles of 9 min self-paced cycling and 1 min sprint. During the cycling trial, your skin temperature, heart rate, oxygen consumption, power output, and cerebral and muscle oxygenation will be continuously monitored. You will be requested to void before commencement of the cycling familiarisation. A urine sample will be collected and nude body weight will be measured. During the trial, you will provide your ratings of perceived exertion after each sprint, and nude body weight will be measured immediately after the cycling trial to calculate sweat volume. After a rest period of at least a week, you will return to the lab for the first of two experimental cycling trials. You will perform the cycling trial once in a thermoneutral environment and once in a hot environment. In addition to the measurements described above in the familiarisation trial, additional measures include core temperature and blood draws. You will also perform the maximal voluntary contractions immediately after the cycling trial, and again at 4 and 24 h and 1 cycle of the cycling trial at these time points. After a rest period of at least a week, you will return to perform the experimental trial in the other environmental condition. Following the experimental trials, you will embark on a 12-week exercise training programme. Upon completion of the exercise training programme, you will perform the experimental cycling trials in both the thermoneutral and hot environments as described above. Thus, you will undertake a total of four 40-min time trials and eight 10-min time trials.

## 5 Your Responsibilities In This Study

You will be required to follow the instructions of the investigators and attend all testing sessions which includes a baseline session, a familiarisation session, and four experimental cycling trials. The baseline and familiarisation sessions are expected to take up to 4 h and the experimental cycling trials are expected to take up to 8 h and another 2 h on the following day. Training sessions will take place over 12 weeks, 3 times per week, 1 h per session. You will

also be required to abstain from strenuous physical activity and caffeine and alcohol ingestion for 48 h before each trial, and from all products containing ephedrine for the duration of your involvement in this study.

## 6 Possible Benefits From Participating In This Research

You will receive data collected from your own measurements and performance. Measurements from dual x-ray absorptiometry can provide information such as body composition and lean limb mass. All exercise training sessions will be fully supervised and personalised. Participation in this research is completely voluntary and at no cost to you. You will be invited to a presentation upon completion of the entire research to share the findings.

## 7 Possible Risks and Side Effects

During the maximal voluntary contractions and cycling trials, you will be instrumented with two pairs of laser probes to monitor absorption of light across cerebral and muscle tissue. There is a risk that the lasers will be directed at the participant’s eyes and cause damage. To minimise this risk, the probes will be turned on only when they are secured in position.

The potential side effects of magnetic stimulation include temporary loss of hearing, seizures, syncope, and headaches. Contraindications include inducing malfunctioning of implanted devices such as cochlear implants or medication pumps. A questionnaire will be used to assess your suitability for this research.

Possible side effects of blood sampling include faintness, bruising, pain, inflammation of the vein or bleeding at the site of the puncture. You will be given instructions to minimise bruising. The potential of bruising at the site is not health deleterious and will clear in a few days.

The DXA assessment involves exposure to a small amount of radiation. As part of everyday living, everyone is exposed to naturally occurring background radiation and receives a dose of about 2,000 microsieverts (uSv) each year. The effective dose from this DXA assessment is 1 uSv. At this dose level, no harmful effects of radiation have been demonstrated as any effect is too small to measure. Thus, according to the *Australia Radiation Protection and Nuclear Safety Agency* the level of risk in this project is considered minimal and is equivalent to Risk Category I (<1:100,000). Do retain this information sheet for at least five years so that it can be provided to staff in any future activities involving exposure to ionizing radiation.

In order to reduce the risks and to enhance safety during the conduct of this research, individuals displaying any of the criteria below will be excluded from the study:

- Smokers
- Existing medical problems
- On medications known to influence motor cortex excitability, such as anti-depressants, benzodiazepines, neuroleptics, and anticonvulsants.

## 8 Voluntary Participation

Your participation in this research is purely voluntary. Trials will cease pre-maturely if you request to stop or when the investigators deem that the trial should be terminated. You have the right to withdraw from this research at any time, without penalty.

## 9 Confidentiality

All data collected will remain completely confidential. Photography and videography of the research may be carried out for documentation purposes. Only the investigators will have access to the data, photographs, and videos. All information collected may be used for publications in peer reviewed journals, conferences, and a thesis in a manner that does not reveal your identity.

## 10 Who To Contact If You Have Questions

Charles Sturt University's Human Research Ethics Committee has approved this study.

If you have any complaints or concerns about this research, you may contact:

**Executive Officer**
**Human Research Ethics Committee**
**Office of Academic Governance**
**Charles Sturt University**
**Panorama Avenue**
**Bathurst NSW 2795**

Tel: +612 6338 4628

Email: ethics@csu.edu.au

As we are looking to recruit volunteers to participate in the above research, we hope this information gives you a brief overview of the research that we are conducting. Thank you for taking the time to read this information sheet and we look forward to your participation in this research. If you have any questions or concerns, please feel free to contact the investigators listed in Section 2.
